# Temporal Cross-Correlations between Ambient Air Pollutants and Seasonality of Tuberculosis: A Time-Series Analysis

**DOI:** 10.3390/ijerph16091585

**Published:** 2019-05-06

**Authors:** Hua Wang, Changwei Tian, Wenming Wang, Xiaoming Luo

**Affiliations:** 1 Department of Infectious Disease Control, Kunshan Centers for Disease Control and Prevention, Kunshan 215300, China; wanghuaksjk@163.com (H.W.); wangwmksjk@163.com (W.W.); 2 Department of Public Health, Soochow University, Kunshan 215300, China

**Keywords:** ambient air pollutants, cross-correlogram, generalized additive model, tuberculosis

## Abstract

The associations between ambient air pollutants and tuberculosis seasonality are unclear. We assessed the temporal cross-correlations between ambient air pollutants and tuberculosis seasonality. Monthly tuberculosis incidence data and ambient air pollutants (PM_2.5_, PM_10_, carbon monoxide (CO), nitrogen dioxide (NO_2_), ozone (O_3_), sulfur dioxide (SO_2_)) and air quality index (AQI) from 2013 to 2017 in Shanghai were included. A cross-correlogram and generalized additive model were used. A 4-month delayed effect of PM_2.5_ (0.55), PM_10_ (0.52), SO_2_ (0.47), NO_2_ (0.40), CO (0.39), and AQI (0.45), and a 6-month delayed effect of O_3_ (−0.38) on the incidence of tuberculosis were found. The number of tuberculosis cases increased by 8%, 4%, 18%, and 14% for a 10 μg/m^3^ increment in PM_2.5_, PM_10_, SO_2_, and NO_2_; 4% for a 10 unit increment in AQI; 8% for a 0.1 mg/m^3^ increment in CO; and decreased by 4% for a 10 μg/m^3^ increment in O_3_. PM_2.5_ concentrations above 50 μg/m^3^, 70 μg/m^3^ for PM_10_, 16 μg/m^3^ for SO_2_, 47 μg/m^3^ for NO_2_, 0.85 mg/m^3^ for CO, and 85 for AQI, and O_3_ concentrations lower than 95 μg/m^3^ were positively associated with the incidence of tuberculosis. Ambient air pollutants were correlated with tuberculosis seasonality. However, this sort of study cannot prove causality.

## 1. Introduction

Tuberculosis is an infectious disease caused by the bacillus, *Mycobacterium tuberculosis*. It typically affects the lungs (pulmonary tuberculosis), but can also affect other sites (extrapulmonary tuberculosis). The disease is spread when people who are sick with pulmonary tuberculosis expel bacteria into the air, for example, by coughing. Persons infected with *Mycobacterium tuberculosis* may develop latent tuberculosis infection, and about 10% of people with latent tuberculosis infection subsequently progress to active tuberculosis themselves [1]. The risk of progression to active disease is highest in the first two years after infection, but persists for life unless treated. The probability of developing tuberculosis disease is much higher among people infected with HIV, and also higher among people affected by risk factors, such as under-nutrition, diabetes, smoking, and alcohol consumption [2]. Without treatment, about 70% of individuals with sputum smear-positive pulmonary tuberculosis die within 10 years of being diagnosed, as do about 20% of people with culture-positive (but smear-negative) pulmonary tuberculosis [2].

Globally, the estimated years of life lost with tuberculosis was 407,188,000 in 2016 [3]. To reach the first milestones of the End Tuberculosis Strategy, the tuberculosis incidence is anticipated to fall at 4% to 5% per year, while it is falling at about 2% per year worldwide (http://www.who.int/tb/publications/global_report/en/, accessed on 15 March 2019). China is one of the 30 tuberculosis high burden countries, and the incidence of tuberculosis ranks second among all of the notifiable diseases in China (http://www.nhc.gov.cn/, accessed on 15 March 2019). Identification of the predictors or risk factors of tuberculosis is crucial for tuberculosis prevention and control. Tuberculosis is a seasonal disease worldwide [4,5]. Globally, a seasonal pattern of tuberculosis with a mostly predominant peak is seen during the spring and summer seasons, which leads to the assumption that the risk of transmission of *Mycobacterium tuberculosis* does appear to be the greatest during winter months [4]. There are several possible reasons of the seasonality of tuberculosis: Serum vitamin D level variability, indoor activities, seasonal changes in immune function, and patient or health care system delays in the diagnosis and treatment of tuberculosis [4]. Additionally, seasonal variation in food availability and food intake, age, and sex are important factors which can play a role in tuberculosis notification variability [4]. While there is consistent evidence that indoor air pollution exposure is associated with an increased risk of tuberculosis [6], a few recent observational studies with mixed findings investigated the effects of ambient air pollutants on the seasonality or risk of tuberculosis [7,8,9]. The highest rate of a major pollutant over China was particulate matter (PM) of PM_2.5_ followed by PM_10_, O_3_, NO_2_, SO_2_, and CO [10], and in 2014 (2015, 2016), 7% (14%, 19%), 17% (27%, 34%), 51% (67%, 70%), and 88% (97%, 98%) of the population in China lived in areas that meet the level of annual PM_2.5_, PM_10_, NO_2_, and SO_2_ standard metrics from Chinese Ambient Air Quality Standards-Grade II [11]. Therefore, even small health effects of ambient air pollutants on the risk of tuberculosis could have considerable public health consequences considering the population exposure in China. However, to our knowledge, there is only one study involving a city in mainland China suggesting that the outdoor PM_2.5_ concentration could be a potential risk factor for the seasonality of tuberculosis [7], and the effects of other major ambient air pollutants (PM_10_, O_3_, NO_2_, SO_2_, and CO) and the air quality index (AQI) on the seasonality of tuberculosis have not been assessed in mainland China. Therefore, with the hypotheses that ambient air pollutants may be associated with the seasonality of tuberculosis, we conducted a time-series analysis to assess the temporal cross-correlations between ambient air pollutants (PM_2.5_, PM_10_, O_3_, NO_2_, SO_2_, and CO) and the air quality index (AQI) and the seasonality of tuberculosis based on the monthly incidence data of tuberculosis and ambient air pollution data in Shanghai from 2013 to 2017.

## 2. Materials and Methods

### 2.1. Data Collection

Serving as the center of economy and finance of China, Shanghai (31°53′ N/122°12′ E) is located in eastern China. According to the China Statistical Yearbook in 2017 (http://www.stats.gov.cn/, accessed on 15 March 2019), Shanghai GDP per capita (17,548 dollars) ranks second (after Beijing) among the 31 provinces in China, and the resident population in Shanghai is 24,197,000 in 2017. According to the Shanghai Statistical Yearbook (http://www.stats-sh.gov.cn/html/sjfb/201901/1003014.html, accessed on 15 March 2019), the population in Shanghai has been relatively stable, and the net growth rates of the population accounting for immigration and emigration were 0.553% in 2011, 0.397% in 2012, 0.424% in 2013, 0.403% in 2014, 0.437% in 2015, and 0.457% in 2016. During January 2008 to December 2011, the mean daily temperature was 17.3 °C (range, −3.4 to 35.7 °C), reflecting the subtropical climate in Shanghai [12]. In addition, the mean relative humidity was 69% (range, 23% to 95%), and the precipitation was 30.9 mm (range, 0 to 1284.0 mm), and the wind speed was 2.9 m/s (range, 0.8 to 7.3 m/s) [12]. In addition, during January 2013 to December 2014, the mean daily temperature was 15.0 °C (range, −4.2 to 31.3 °C), and the mean relative humidity was 71.3% (range, 31.8% to 97.4%) [13].

The monthly incidence data of tuberculosis are released by the Shanghai Commission of Health and Family Planning, and the monthly incidence data from 2013 to 2017 (i.e., 60 months) were included in this analysis. In China, all tuberculosis cases verified by clinical or laboratory diagnosis must be reported within 24 hours, and then must be checked by professionals from local centers for disease control and prevention. Duplicate cards from the same case must be checked and addressed by the end of each month.

Ambient air pollution data from 2013 to 2017 are obtained by the Shanghai Environmental Monitoring Center, including concentrations of particulate matter of PM_2.5_ (μg/m^3^) and PM_10_ (μg/m^3^), carbon monoxide (CO) (mg/m^3^), nitrogen dioxide (NO_2_) (μg/m^3^), ozone (O_3_) (μg/m^3^), sulfur dioxide (SO_2_) (μg/m^3^), and the air quality index (AQI), which considers PM_2.5_, PM_10_, CO, NO_2_, O_3_, and SO_2_ simultaneously. Daily values of air pollution data were averaged to monthly figures (i.e., 60 months) in this analysis, and then moving averages ranging from 1 to 12 months prior to the current month were calculated. The Shanghai Environmental Monitoring Center is the government agency in charge of the collection of air pollution data in Shanghai. The database from the Shanghai Environmental Monitoring Center included 24 h average measurements of PM_2.5_, PM_10_, CO, NO_2_, and SO_2_ concentrations, as well as the daily maximum O_3_ figure based on an 8 h running mean. All data used in this study were based on the average assessment of nine air quality monitoring stations in Shanghai, located in Putuo, Yangpu, Huangpu, Qingpu, Hongkou, Xuhui, Jing’an, and Pudong New Area, and all data were covered by China National Quality Control [14]. These monitoring stations are mandated to be located away from major roads, industrial sources, buildings, or residential sources of emissions from the burning of coal, waste, or oil; thus, these monitoring results reflect the background urban air pollution level in Shanghai rather than local sources, such as traffic or industrial combustion [13,14]. Ethical approval is not required for this study because these are secondary data for public access.

### 2.2. Statistical Analysis

In the descriptive analysis, scatter plots of both the exposure (PM_2.5_, PM_10_, CO, NO_2_, O_3_, SO_2_, and AQI) and the number of tuberculosis cases over time for the entire study period (2013–2017) were provided to reveal high-level patterns in the data. Cross-correlograms help to explore relationships between two time series [15]. To assess the effects of ambient air pollutants on the risk of tuberculosis, we first conducted cross-correlation analysis over a range of time lags. We listed the ambient air pollutants fist and then the tuberculosis incidence data second in the analysis, then the positive lags denote correlations between ambient air pollutants at time t and the incidence of tuberculosis at time t + 1, t + 2, etc. [10]. Both the concentrations from a single month and moving average concentrations of PM_2.5_, PM_10_, CO, NO_2_, O_3_, SO_2_, and AQI were used in this analysis. Of those infected, approximately 90% will be latently infected, 5% will progress to active tuberculosis within one year, and an additional 5% will progress to active tuberculosis within their lifetime [16,17]. There is also evidence showing that recent tuberculosis transmission is more influenced by the season than tuberculosis resulting from activation of a latent infection [18]. Therefore, the number of lags was defined to be 0 to 2 months in this analysis.

Generalized additive models (GAM) are flexible extensions of generalized linear models. Whereas the link function in a generalized linear models is a linear combination of the predictors, in a GAM, the link function may include flexible functions of arbitrary complexity based (for example) on smoothing splines. GAM is useful for exploratory analysis when one knows very little about the functional forms in a dataset, such as the relationship between ambient air pollutants and influenza like-illness [19,20]. In this analysis, cubic regression spline functions of nonlinear terms (df = 3), including PM_2.5_, PM_10_, CO, NO_2_, O_3_, SO_2_, and AQI one at a time, were incorporated into the generalized additive models. The number of tuberculosis cases per month is assumed to follow a Poisson distribution [5], thus Poisson regression analysis was used in GAM, and the most commonly used log link function was adopted for ease of interpretation [19]. To explore the delayed impact of ambient air pollutants on the risk of tuberculosis, GAM was conducted with ambient air pollutants that lagged at a single month (0–12 months) or moving average (2–12 months), ranging from 0 to 4 months prior to the tuberculosis notification date. Flexible spline functions were adopted to allow control of seasonality and long-term trends [21]. To fit a spline function in practice, we first generated a set of basis variables, which are functions of the main time variable, and then included these basis variables in the Poisson model. Relative risk and 95% confidence intervals (IRR (95%CI)) from the GAM model were provided. All analysis was conducted with Stata version 10.0, with *p*-values < 0.05 considered significant.

## 3. Results

The mean concentration (standard deviation) of PM_2.5_ was 49.95 (18.85) μg/m^3^, and the values were 67.44 (22.38) μg/m^3^ for PM_10_, 16.85 (8.15) μg/m^3^ for SO_2_, 45.38 (13.00) μg/m^3^ for NO_2_, 0.80 (0.16) mg/m^3^ for CO, 103.99 (28.84) μg/m^3^ for O_3_, and 86.35 (18.27) for AQI. Supplementary Figure S1 shows scatter plots of both the exposures and number of tuberculosis cases over time for the entire study period, and seasonality and trends with the flexible cubic spline model are also shown in Supplementary Figure S1. The plots show that both the ambient air pollutants and the number of tuberculosis cases seem to be dominated by annual seasonal patterns, with PM_2.5_, PM_10_, CO, NO_2_, SO_2_, and AQI highest in winter and lowest in summer, and the opposite pattern for O_3_, while the highest number of tuberculosis cases was observed in spring–summer (May, June, July, and August) and was lowest in winter. The magnitude of cross-correlation coefficients between PM_2.5_, PM_10_, SO_2_, NO_2_, CO, and AQI increased with prolonged lagged time from 0 to 4 months and then decreased thereafter. The 4-month lagged cross-correlation coefficients of PM_2.5_, PM_10_, SO_2_, NO_2_, CO, and AQI with the seasonality of tuberculosis were 0.55, 0.52, 0.47, 0.40, 0.39, and 0.45, respectively. In addition, while a cross-correlation coefficient of 0.41 was observed between the 0 to 1 month lag of O_3_ and tuberculosis, the magnitude of the cross-correlation coefficients increased to −0.38 at the 6-month lag of O_3_ and then decreased thereafter (Table 1).

**Figure 1:**
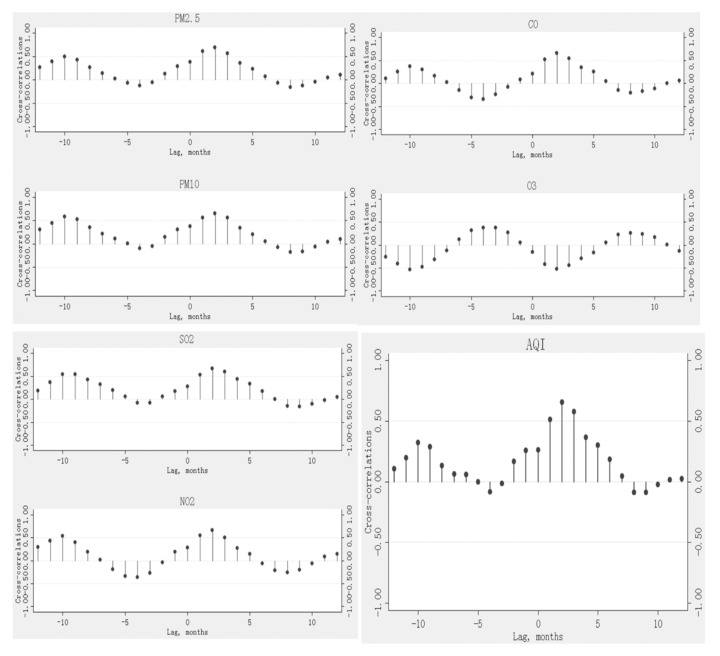
Cross-correlograms between 6-month moving average concentrations of PM_2.5_, PM_10_, SO_2_, and AQI and the seasonality of tuberculosis, and cross-correlograms between 7-month moving average concentrations of NO_2_, CO, and O_3_ and the seasonality of tuberculosis.

The largest magnitude cross-correlation coefficients were found between 6-month moving average concentrations of PM_2.5_, PM_10_, SO_2_, and AQI and the seasonality of tuberculosis two months later, and the cross-correlation coefficients were 0.69, 0.65, 0.67, and 0.66, respectively. Largest magnitude cross-correlation coefficients were found between 7-month moving average concentrations of NO_2_, CO, and O_3_ and the seasonality of tuberculosis two months later, and the cross-correlation coefficients were 0.67, 0.66, and −0.52, respectively. In addition, the relatively strong positive cross-correlations between O_3_ lagged at a single month (0–1 month) and the seasonality of tuberculosis were not observed in the analysis with moving average concentrations (Table 2). The above cross-correlograms are shown in Figure 1.

**Figure 2:**
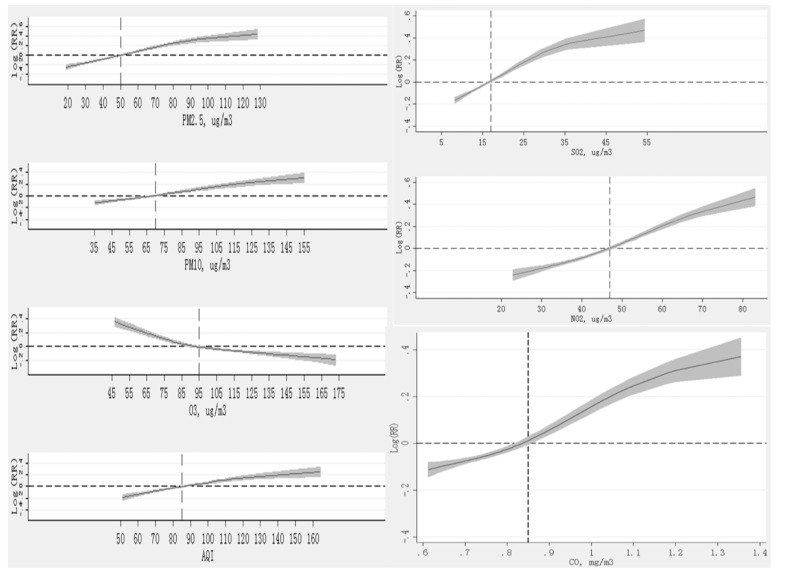
The non-linear relationships between concentrations of PM_2.5_, PM_10_, SO_2_, NO_2_, CO, O_3_, and AQI and the risk of tuberculosis after controlling for seasonality and long-term trends. The solid lines indicate the estimated log relative risk of tuberculosis and the dashed lines indicate the corresponding 95% confidence intervals.

The non-linear relationships (all *p*-values for non-linear tests are <0.01) between ambient air pollutants and the risk of tuberculosis after controlling for seasonality and long-term trends are shown in Figure 2. PM_2.5_ concentrations above 50 μg/m^3^, 70 μg/m^3^ for PM_10_, 16 μg/m^3^ for SO_2_, 47 μg/m^3^ for NO_2_, 0.85 mg/m^3^ for CO, and 85 for AQI, and O_3_ concentrations lower than 95 μg/m^3^ were significantly positively associated with the incidence of tuberculosis. The departure from a linear relationship may be mainly caused by the relatively large number of tuberculosis cases included in the model (i.e., the pointwise 95% confidence intervals are very narrow). After controlling for seasonality and long-term trends, the number of tuberculosis cases increased by 8% (RR (95% CI): 1.08 (1.05–1.10), *p* = 0.01), 4% (1.04 (1.01–1.06), *p* = 0.03), 18% (1.18 (1.10–1.26), *p* < 0.01), and 14% (1.14 (1.10–1.19), *p <* 0.01) for a 10 μg/m^3^ increment in PM_2.5_, PM_10_, SO_2_, and NO_2_; and 4% (1.04 (1.01–1.06), *p* = 0.03) for a 10 unit increment in AQI; 8% (1.08 (1.04–1.11), *p* < 0.01) for a 0.1 mg/m^3^ increment in CO; and decreased by 4% (0.96 (0.92–0.99), *p* = 0.04) for a 10 μg/m^3^ increment in O_3_. Similar results were found in the analysis of moving average concentrations (Supplementary Figure S2).

## 4. Discussion

To our knowledge, this is the first time series analysis investigating the cross-correlations and non-linear relationships of ambient air pollutants with the seasonality of tuberculosis in China. In this study, ambient air pollutants of PM_2.5_, PM_10_, SO_2_, NO_2,_ CO, and AQI were found to be positively associated with the seasonality of tuberculosis, while O_3_ was inversely associated with the seasonality of tuberculosis, and delayed effects of ambient air pollutants on the incidence of tuberculosis were found. Moving average concentrations of ambient air pollutants yielded better predictive power than concentrations of pollutants from a single month. Furthermore, threshold effects of ambient air pollutants on the incidence of tuberculosis were detected.

The effects of ambient air pollutants on the risk or seasonality of tuberculosis have been investigated in a few studies with mixed findings. An ecologic study conducted in Beijing and Hong Kong suggested that outdoor PM_2.5_ concentration could be a potential risk factor for the seasonality of tuberculosis [7], and a 10 mg/m^3^ increase in PM_2.5_ concentrations was significantly associated with a 3% increase in the number of tuberculosis cases [7]. A cohort study with a median follow-up of 6.7 years in Taiwan found that the risk of active tuberculosis was 1.39 (0.95–2.03) and 0.95 (0.78–1.17) with a 10 μg/m^3^ increase in PM_2.5_ and PM_10_; 1.33 (1.04–1.70), 1.21 (1.04–1.41), and 0.69 (0.48–0.98) with a 10 ppb increase in NO_2_, nitrogen oxides, and O_3_; and 1.89 (0.78–4.58) with a 10 ppm increase in CO, respectively [8]. In a nested case-control study in the USA, tuberculosis was found to be positively associated with ambient CO (highest vs. lowest quintile: 1.50 (1.15–1.95)) and NO_2_ (1.42 (1.10–1.84)), while no association was found with PM_2.5_, PM_10_, SO_2_, and O_3_ [9]. An ecological study in the USA indicated a potential association between PM_2.5_ and PM_10_ and tuberculosis [22]. A retrospective cohort study in South Korea found no associations between PM_10_, CO, NO_2_, and O_3_ and tuberculosis, while the interquartile increase in the SO_2_ concentration was associated with a 7% increment in tuberculosis incidence [23]. A significant correlation of smear-positive tuberculosis and PM_2.5_ was found after a retrospective medical records review, but no association was found with O_3_ in a USA study [24]. Tuberculosis showed a highly significant correlation with PM_2.5_ in a cross-sectional study in Japan [20]. In addition, exposure to PM_2.5_ [25] and residential proximity to road traffic volumes and traffic density [26] were also found to increase the risk of death in tuberculosis patients.

Approximately 20% of the individuals who come into close contact with patients with smear-positive tuberculosis will subsequently be infected. Of those infected, approximately 90% will be latently infected, 5% will progress to active tuberculosis within one year, and an additional 5% will progress to active tuberculosis within their lifetime [16,17]. In this study, pollutants of PM_2.5_, PM_10_, SO_2_, NO_2_, CO, and AQI were associated with an increased number of tuberculosis notification four months later. In China, monthly changes in both the air pollution ratio and continuous air pollution ratio has a U-shaped variation, indicating that the highest levels of air pollution occur in winter and the lowest levels happen in summer [27]. A recent review showed that the inter-quartile range of tuberculosis diagnostic delay was 44 to 77.8 days in low- and middle-income countries [28], and there is also evidence showing that recent tuberculosis transmission is more influenced by season than tuberculosis resulting from activation of a latent infection [18]. Therefore, the lagged effects in this study are consistent with the evidence available given the preclinical period, from infection to development of active tuberculosis, may last from a few weeks to several months [4].

Particulate matter may modulate the innate immune system and increase susceptibility to infection through (a) alveolar macrophage-driven inflammation, recruitment of neutrophils, and disruption of barrier defenses; (b) alterations in alveolar macrophage phagocytosis and intracellular killing; and (c) increased susceptibility to infection via upregulation of receptors involved in pathogen invasion [29]. In addition, a recent study found that particulate matter could adsorb antimicrobial proteins and peptides and create negative complexes, thereby decreasing the functional amount of antimicrobial proteins and peptides capable of killing pathogens [30]. Furthermore, recent findings also showed that the mixture of components in ambient air particulate matter may explain some seasonal differences in associations between health outcomes and particulate matter in epidemiologic studies by their pro-inflammatory potential [31]. The detailed mechanisms underlying other ambient air pollutants of NO_2_, SO_2_, and O_3_ and tuberculosis warrant further investigation.

There are several limitations. First, this analysis aimed to explore the temporal cross-correlations between ambient air pollutants and seasonality of tuberculosis, and causality cannot be confirmed by this type of analysis. Second, detailed information of tuberculosis cases are missing, such as age and sex, which precludes further analysis in this study. Third, meteorological factors that may improve the GAM were not available to us. Fourth, although all tuberculosis cases verified by the clinical or laboratory diagnosis must be reported within 24 hours in China, non-notified cases who do not seek health care may also influence our results. Fifth, tuberculosis is generally associated with poverty and deprivation, and household crowding is one manifestation of poverty and could be a mechanism that mediates the link between deprivation and tuberculosis [1]. Reducing levels of household crowding is likely to be most important for those populations with both high rates of tuberculosis and high rates of household crowding [1]. Therefore, further analysis incorporating indoor air quality, and meteorological (temperature, relative humidity, wind, and rain), social, and economic parameters is warranted, with the identification of high-risk groups. Finally, tuberculosis incidence data are released each month, thus we did not explore the short-term associations that could be modeled with the distributed lag models [21,32]. However, for an outcome, like tuberculosis, the period from latent tuberculosis infection to active tuberculosis usually lasts for several months or longer. Therefore, in this analysis, we used the cross-correlograms to explore the long-term cross-correlations.

## 5. Conclusions

In summary, ambient air pollutants of PM_2.5_, PM_10_, SO_2_, NO_2_, CO, O_3_, and AQI were associated with the seasonality of tuberculosis in Shanghai, China. The findings need to be confirmed in other provinces in China, and also in other countries. In addition, this sort of study cannot prove causality and several other factors, including indoor air quality, and meteorological (temperature, relative humidity, wind, and rain), social, and economic parameters are not available in this analysis, which should be considered in further studies. Overall, this study marks the beginning of deeper investigations and more research is needed before any substantial recommendations can be made for environmental policy.

## Figures and Tables

**Table 1:** Cross-correlation coefficients between concentrations of PM_2.5_, PM_10_, SO_2_, NO_2_, CO, O_3_, and air quality index (AQI) at a single month over a range of time lags (0–12 months) and seasonality of tuberculosis.

Lags	PM_2.5_	PM_10_	SO_2_	NO_2_	CO	O_3_	AQI
	Corr	Corr	Corr	Corr	Corr	Corr	Corr
0	−0.03	0.02	−0.04	−0.17	−0.21	0.41	0.08
1	−0.12	−0.08	−0.18	−0.29	−0.37	0.41	−0.11
2	0.03	−0.03	−0.11	−0.22	−0.29	0.28	−0.05
3	0.35	0.26	0.25	0.15	0.10	−0.04	0.22
4	0.55	0.52	0.47	0.40	0.39	−0.23	0.45
5	0.45	0.44	0.40	0.40	0.33	−0.29	0.30
6	0.37	0.32	0.38	0.38	0.34	−0.38	0.21
7	0.26	0.24	0.37	0.26	0.34	−0.35	0.17
8	0.13	0.18	0.27	0.17	0.19	−0.13	0.22
9	−0.05	−0.01	0.10	−0.02	0.01	−0.00	0.05
10	−0.23	−0.22	−0.15	−0.31	−0.20	0.15	−0.16
11	−0.19	−0.18	−0.17	−0.30	−0.23	0.32	−0.06
12	−0.15	−0.12	−0.15	−0.17	−0.19	0.29	−0.04

**Table 2:** Cross-correlation coefficients between moving average (MV) concentrations ranging from 2 to 12 months of PM_2.5_, PM_10_, SO_2_, NO_2_, CO, O_3_, and AQI over a range of time lags (0–4) and the seasonality of tuberculosis.

Lag (Months)	MV2	MV3	MV4	MV5	MV6	MV7	MV8	MV9	MV10	MV11	MV12
	Corr	Corr	Corr	Corr	Corr	Corr	Corr	Corr	Corr	Corr	Corr
PM_2.5_											
0	−0.08	0.06	0.22	0.32	0.39	0.43	0.44	0.42	0.37	0.33	0.30
1	0.22	0.41	0.51	0.60	0.62	0.63	0.61	0.57	0.54	0.51	0.48
2	0.53	0.61	0.66	0.68	0.69	0.66	0.61	0.58	0.56	0.53	0.51
3	0.57	0.60	0.61	0.60	0.57	0.51	0.47	0.45	0.42	0.41	0.44
4	0.46	0.47	0.46	0.42	0.36	0.32	0.30	0.27	0.27	0.31	0.35
PM_10_											
0	−0.08	0.03	0.20	0.31	0.38	0.42	0.45	0.43	0.38	0.34	0.31
1	0.14	0.34	0.46	0.52	0.56	0.59	0.58	0.52	0.48	0.46	0.42
2	0.47	0.56	0.60	0.63	0.65	0.63	0.57	0.53	0.51	0.48	0.45
3	0.56	0.57	0.58	0.59	0.56	0.49	0.45	0.43	0.39	0.37	0.39
4	0.44	0.45	0.46	0.42	0.34	0.30	0.28	0.24	0.23	0.25	0.30
SO_2_											
0	−0.19	−0.06	0.10	0.20	0.27	0.34	0.39	0.40	0.36	0.33	0.30
1	0.09	0.28	0.38	0.46	0.53	0.58	0.60	0.56	0.53	0.51	0.46
2	0.43	0.51	0.57	0.63	0.67	0.67	0.64	0.61	0.58	0.53	0.50
3	0.50	0.54	0.59	0.61	0.61	0.56	0.52	0.50	0.45	0.42	0.43
4	0.44	0.49	0.51	0.50	0.44	0.40	0.37	0.32	0.29	0.32	0.35
NO_2_											
0	−0.32	−0.19	−0.02	0.11	0.21	0.29	0.34	0.35	0.27	0.19	0.14
1	−0.02	0.17	0.31	0.41	0.49	0.55	0.58	0.53	0.48	0.46	0.40
2	0.35	0.46	0.54	0.60	0.64	0.66	0.61	0.58	0.58	0.54	0.51
3	0.48	0.54	0.56	0.59	0.57	0.50	0.46	0.45	0.42	0.41	0.45
4	0.46	0.46	0.47	0.43	0.34	0.28	0.26	0.21	0.20	0.27	0.34
CO											
0	−0.41	−0.27	−0.10	0.02	0.11	0.20	0.25	0.26	0.21	0.16	0.12
1	−0.08	0.13	0.25	0.36	0.46	0.52	0.54	0.51	0.47	0.44	0.38
2	0.32	0.41	0.50	0.59	0.64	0.65	0.62	0.59	0.57	0.52	0.47
3	0.43	0.49	0.56	0.59	0.59	0.54	0.50	0.48	0.43	0.39	0.42
4	0.39	0.45	0.47	0.45	0.40	0.35	0.32	0.27	0.24	0.27	0.33
O_3_											
0	0.40	0.28	0.16	0.05	−0.06	−0.15	−0.20	−0.21	−0.18	−0.09	−0.01
1	0.12	−0.03	−0.14	−0.26	−0.36	−0.42	−0.45	−0.45	−0.41	−0.36	−0.28
2	−0.18	−0.27	−0.38	−0.46	−0.50	−0.52	−0.52	−0.49	−0.46	−0.41	−0.37
3	−0.31	−0.39	−0.46	−0.47	−0.47	−0.45	−0.40	−0.37	−0.32	−0.30	−0.34
4	−0.38	−0.43	−0.42	−0.40	−0.36	−0.29	−0.25	−0.20	−0.17	−0.23	−0.30
AQI											
0	−0.13	−0.03	0.14	0.23	0.26	0.29	0.32	0.32	0.27	0.25	0.24
1	0.11	0.33	0.43	0.49	0.51	0.56	0.55	0.51	0.49	0.48	0.44
2	0.44	0.53	0.58	0.62	0.66	0.65	0.59	0.57	0.56	0.53	0.50
3	0.48	0.50	0.54	0.59	0.58	0.51	0.48	0.47	0.43	0.41	0.43
4	0.32	0.36	0.43	0.43	0.36	0.34	0.33	0.29	0.27	0.30	0.34

MV: moving average.

## Supplementary Materials

The following are available online at https://www.mdpi.com/1660-4601/16/9/1585/s1, Figure S1: title, Table S1: title, Video S1: title. Figure S1: Seasonal and long-term patterns of outcome (TB cases) and exposure (PM_2.5_, PM_10_, O_3_, SO_2_, NO_2_, CO, AQI) data over time. Gray circle: raw data, solid black line: flexible cubic spline model. Figure S2: The non-linear relationships between 6-month moving average concentrations of PM_2.5_, PM_10_, SO_2_ and AQI, as well as 7-month moving average concentrations of NO_2_, CO and O_3_ and risk of tuberculosis. The solid lines indicate the estimated log relative risk of tuberculosis and the dashed lines indicate the corresponding 95 % confidence intervals.
