# Supplementary figures and images for: Temporal Cross-Correlations between Ambient Air Pollutants and Seasonality of Tuberculosis: A Time-Series Analysis
Source: Int J Environ Res Public Health. 2019 May 6;16(9):1585. doi: 10.3390/ijerph16091585 (PMC6540206; doi:10.3390/ijerph16091585)

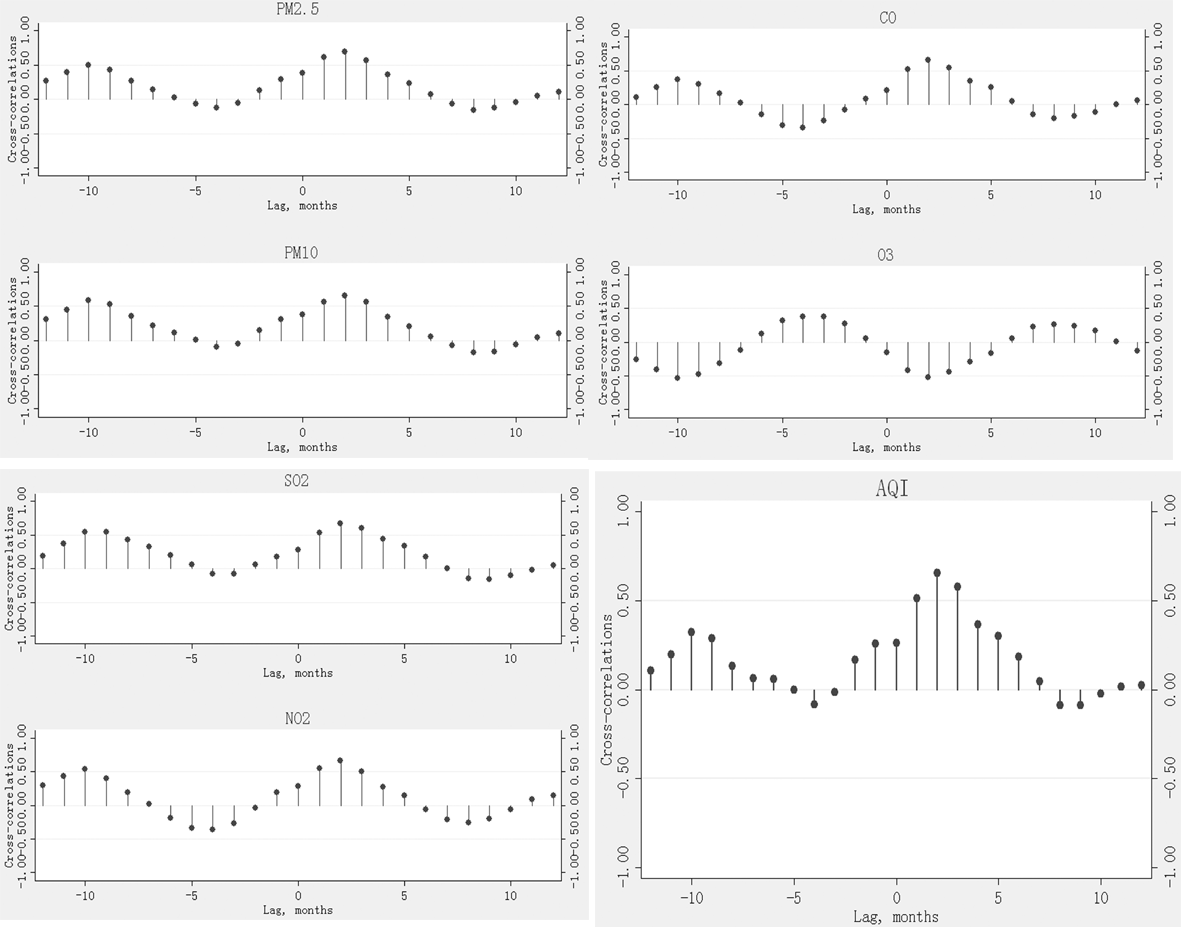

Supplement: Supplementary file 1 [file ijerph-16-01585-s001.zip › Figure 1-2/Figure 1.tif]

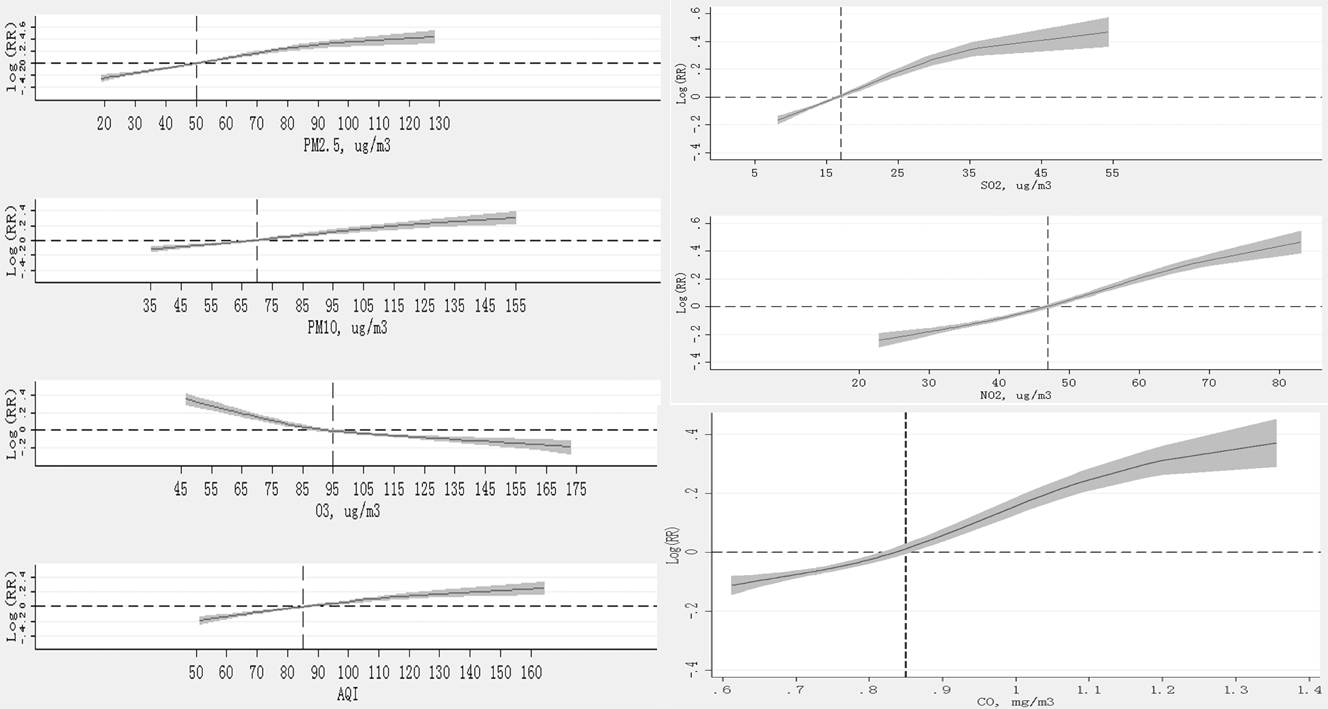

Supplement: Supplementary file 1 [file ijerph-16-01585-s001.zip › Figure 1-2/Figure 2.tif]
